# Editorial: Investigating the Factors that Control Epithelial Cell Polarity and Adhesion During Morphogenesis

**DOI:** 10.3389/fcell.2021.802568

**Published:** 2021-11-18

**Authors:** Lorenza González-Mariscal

**Affiliations:** Department of Physiology, Biophysics and Neuroscience, Cinvestav, Mexico City, Mexico

**Keywords:** epithelia, cell polarity, cell-cell adhesion, morphogenesis, differentiation

In the first part of this research topic, two articles employ *Drosophila* as their models to analyze factors that control epithelial morphogenesis. One analyzes the flow of information from E-cadherin mediated cell-cell contacts to gene transcription at the nucleus, while the other studies the role of a microtubule-binding protein in the morphogenesis of the abdomen. A third article analyzes apical junction proteins expression during epithelial differentiation in *C. elegans* embryos with mutated pha-1 organogenesis factor. In the second part of this research topic, two articles study epithelial morphogenesis in the placenta. One analyzes the role of a GEF protein in syncytiotrophoblast differentiation in the labyrinth of mice placenta, and the other describes how the expression of a morphogen diminishes in human placentas with preeclampsia.

E-cadherin is the transmembrane protein that mediates homophilic adhesion between neighboring cells at the adherens junction in epithelial cells. Loss of E-cadherin expression is a prerequisite of epithelial to mesenchymal transition and is a distinctive feature of cancerous cells. However, E-cadherin re-expression also constitutes a critical step in cancer progression and metastasis. In this research topic, Ramirez Moreno et al. review the currently known E-cadherin transcriptional activation and inhibition mechanisms. Most importantly proposes a novel mechanism that transfers to the nucleus the information on the amount of E-cadherin present at the cell surface. This mechanism was studied in *Drosophila* and relies on the recruitment of STAT92E transcription factor to the cell surface by E-cadherin and its binding partner, the polarity determinant Par-3. A drop of E-cadherin levels releases STAT92E from the cell borders, which following non-canonical signaling enters the nucleus in a non-phosphorylated state, binds non-histone chromatin protein HP1, and localizes at euchromatin regions, promoting E-cadherin expression. This mechanism that relates non-canonical STAT signaling with E-cadherin expression provides feedback for the flow of information between cell-cell adhesion at the surface and transcriptional activation at the nucleus.

The article by Panzade and Matis in this research topic analyzes the organization of the microtubule network during epithelial morphogenesis. In the experimental model of *Drosophila*, they study the formation of the abdominal epidermis by diploid imaginal cells known as histoblasts. Panzade and Matis analyze the role of Patronin, a microtubule minus-end binding protein, in the organization of non-centrosomal microtubule networks. The results reveal that downregulation of Patronin disrupts the normal development of the abdomen, and an in-depth quantitative analysis shows loss of microtubules and non-centrosomal microtubule organization in migrating histoblasts. These results will interest a broad audience concerned with the mechanisms that regulate microtubule organization during morphogenesis since Patronin belongs to the calmodulin regulated spectrin associated protein (CAMSAP) family also found in vertebrates.

Lehmann and Pohl show that *C. elegans* embryos with the loss-of–function mutation *pha-1*(e2123) manifest defects in epithelial differentiation through an altered expression of apical junction components. Moreover, in wild-type embryos laser cutting of dorso-ventral cell-cell contacts triggers a reversal of elongation that is lost in *pha-1*(e2123) embryos but can be recovered by reduced expression of the apical junction component DLG-1.

Beal et al. analyze the role of the RhoGTPase activator p114RhoGEF on trophoblast morphogenesis in mice placenta. p114RhoGEF is a guanine nucleotide exchange factor present at tight junctions that regulates actomyosin contractility. While p114RhoGEF knock-out (KO) mice display embryonic lethality with defects in the morphogenesis of the labyrinth zone of the placenta, the endothelial-specific KO does not trigger embryonic death, suggesting that p114RhoGEF is crucial for trophoblast differentiation in the labyrinth. Since PKA stabilizes epithelial barriers, and the scaffolding protein AKAP12 anchors PKA, the authors analyzed in human trophoblast BeWo cell line if p114RhoGEF affected AKAP12 expression, finding that p114RhoGEF stimulates AKAP expression and promotes cAMP-induced cell-cell fusion, an essential process for syncytiotrophoblast differentiation and placental development. In addition, they observed the need for p114RhoGEF and AKAP12 for the expression of PKA/CREB-dependent target genes required for the differentiation of the syncytiotrophoblast. In a murine trophoblast stem cell line, p114RhoGEF depletion also induced stress fibers and reduced cell migratory capacity, suggesting that p114RhoGEF determines actomyosin organization and drives migration, which is required to achieve the close proximity of neighboring plasma membranes that enables fusion. This work reveals that p114RhoGEF modulates placental morphogenesis by regulating syncytiotrophoblast differentiation by actomyosin cytoskeleton remodeling and PKA signaling.

The article by Li et al. studies human placentas affected with preeclampsia. This common complication of human pregnancy responsible for a high percentage of maternal and neonatal deaths, and premature births, has been related to trophoblast dysfunction, but its precise etiology remains elusive. Li et al. explore the role of the morphogen Syntaxin2 (STX2), also known as epimorphin, on the placental trophoblast, observing a diminished expression in placentas from patients with preeclampsia compared to those from normal pregnancies. In addition, loss and gain of function experiments done in immortalized and primary human trophoblast cells reveal that STX2 promotes cell proliferation, migration, and invasion, through STX2 interaction with p85, a regulatory subunit of PI3K. Their results suggest that in preeclampsia, STX2 downregulation results in trophoblast dysfunction by inactivating the PI3K-AKT pathway. This work advances our knowledge of preeclampsia pathogenesis and highlights the possibility of employing STX2 as a diagnostic tool or even a therapeutic agent in preeclampsia patients.

